# Graphene Oxide Chemistry Management via the Use of KMnO_4_/K_2_Cr_2_O_7_ Oxidizing Agents

**DOI:** 10.3390/nano11040915

**Published:** 2021-04-03

**Authors:** Kseniya A. Shiyanova, Maksim V. Gudkov, Maxim K. Rabchinskii, Liliia A. Sokura, Dina Y. Stolyarova, Marina V. Baidakova, Dmitriy P. Shashkin, Andrei D. Trofimuk, Dmitry A. Smirnov, Ivan A. Komarov, Victoria A. Timofeeva, Valery P. Melnikov

**Affiliations:** 1 N.N. Semenov Federal Research Center for Chemical Physics, Russian Academy of Sciences, 119991 Moscow, Russia; shiyanovakseniya@mail.ru (K.A.S.); gudkovmv@gmail.com (M.V.G.); icp@chph.ras.ru (D.P.S.); vik.timofeeva@gmail.com (V.A.T.); 2 Ioffe Institute, 194021 Saint Petersburg, Russia; rabchinskii@mail.ioffe.ru (M.K.R.); sokura@mail.ioffe.ru (L.A.S.); baidakova@mail.ioffe.ru (M.V.B.); trofimuk.ad@gmail.com (A.D.T.); 3 NRC “Kurchatov Institute”, 123182 Moscow, Russia; stolyarova.d@gmail.com; 4 Institut für Festkörper- und Materialphysik, Technische Universität Dresden, 01069 Dresden, Germany; dmitry.smirnov@helmholtz-berlin.de; 5 Department of Composite Construction for Space Rockets, Bauman Moscow State Technical University, 105005 Moscow, Russia; master_kom@mail.ru

**Keywords:** graphene oxide, oxidation method, chemical composition management

## Abstract

In this paper, we propose a facile approach to the management of graphene oxide (GO) chemistry via its synthesis using KMnO_4_/K_2_Cr_2_O_7_ oxidizing agents at different ratios. Using Fourier Transformed Infrared Spectroscopy, X-ray Photoelectron Spectroscopy, and X-ray Absorption Spectroscopy, we show that the number of basal-plane and edge-located oxygenic groups can be controllably tuned by altering the KMnO_4_/K_2_Cr_2_O_7_ ratio. The linear two-fold reduction in the number of the hydroxyls and epoxides with the simultaneous three-fold rise in the content of carbonyls and carboxyls is indicated upon the transition from KMnO_4_ to K_2_Cr_2_O_7_ as a predominant oxidizing agent. The effect of the oxidation mixture’s composition on the structure of the synthesized GOs is also comprehensively studied by means of X-ray diffraction, Raman spectroscopy, transmission electron microscopy, atomic-force microscopy, optical microscopy, and the laser diffraction method. The nanoscale corrugation of the GO platelets with the increase of the K_2_Cr_2_O_7_ content is signified, whereas the 10–100 μm lateral size, lamellar, and defect-free structure is demonstrated for all of the synthesized GOs regardless of the KMnO_4_/K_2_Cr_2_O_7_ ratio. The proposed method for the synthesis of GO with the desired chemistry opens up new horizons for the development of graphene-based materials with tunable functional properties.

## 1. Introduction

In recent years, graphene has attracted much attention from both experimental and theoretical research groups due to its unique structural, mechanical, heat-conducting, and electricity-conducting properties [1,2,3,4,5]. This makes graphene attractive for application in various fields, such as microelectronics [6,7,8,9,10], sensors [11,12,13,14,15], biomedicine [16], and the accumulation of electrical energy [17,18], etc.

It should be noted that the ideal graphene structure is not required in all potential applications. An increasing number of studies have been devoted to the synthesis and application of graphenes modified with organic groups and having a defect structure, which—in many publications—are also called ‘functionalized graphenes’ or ‘chemically modified graphenes’ (CMGs) [19,20,21,22]. One of the most important CMGs is graphene oxide (GO), a nonstoichiometric derivative of graphene, the edges and surfaces of which are covered with various oxygen-containing functional groups. The interest in GO is primarily due to the fact that it forms stable dispersions in water and a number of other polar solvents, and can also be reduced by thermal or chemical action to a graphene-like material (reduced graphene oxide, rGO) [23,24,25,26,27] with a given composition of organic groups. By controlling the chemical composition, one can adjust the electronic structure of materials, their sorption capacity, ability to electrical and thermal conductivity, affinity for composite components, and ability to participate in ion exchange reactions and bind to various biological objects (proteins, antibodies, aptamers, etc.). This opens up new opportunities to optimize the physical properties of graphene for practical applications, and to form new graphene-based smart materials.

Brodie, for the first time, synthesized GO by the treatment of graphite with a mixture of HNO_3_ and KClO_3_, with the subsequent product isolation and re-treatment with a reaction mixture several times. The thus-produced GO had low oxidation degree, and was described by the formula C_11_H_4_O_5_ [28]. An application of the Staudenmaier method, based on the use of concentrated H_2_SO_4_ as a reaction medium and an increased amount of KClO3 [29,30], made it possible to achieve a high content of hydroxyl groups (C-OH) with a small amount of the edge-located carbonyls (C=O) [31]. According to the X-ray photoelectron spectroscopy data, the C/O ratio of the resulting product was 2.47. However, the method turned out to be laborious and dangerous: despite only one stage being involved, the addition of potassium chlorate lasted more than 1 week (due to the explosiveness of the reaction mixture), and the released chlorine dioxide had to be removed with an inert gas [29].

The classical method for the synthesis of GO is considered to be the method proposed in 1958 by Hummers and Offeman [32]. The method is based on the application of a concentrated H_2_SO_4_, NaNO_3_, and KMnO_4_ mixture at temperatures below 45 °C. The entire oxidation process is completed within 2 h, and provides a higher oxidation degree (C/O ratio 2.05) than the Staudenmaier method, along with a higher content of C=O groups in the resulting GO [31]. In order to reduce the amount of the unoxidized fraction after graphite oxidation via the Hummers method, Kovtyukhova et al. proposed, in 1999, to pretreat graphite with a mixture of H_2_SO_4_, K_2_S_2_O_8_, and P_2_O_5_ at 80 °C for several hours [33]. Modified Hummers methods are currently the most common way to synthesize GO. The use of one of the most common modifications, the Marcano method [34], leads to a product with an even higher oxidation state (C/O ratio 1.95) compared to other methods. Furthermore, it contains an increased content of C-OH and C=O functional groups, with the appearance of a noticeable amount of carboxyls (COOH) [31]. Other works on various modifications of the Hummers method also suggest an increase in the amount of potassium permanganate, and changes in temperature conditions and processing time, etc. [25,26,27].

In 2010, Chandra et al. [35] proposed an alternative approach for the synthesis of GO by the replacement of KMnO_4_ with K_2_Cr_2_O_7_. Graphite was mixed with NaNO_3_ and concentrated H_2_SO_4_ in an ice bath. Then, K_2_Cr_2_O_7_ was slowly added and kept under stirring for 5 days at room temperature. In 2018, Martin Rosillo-Lopez and Christoph G. Salzmann [36] presented an optimized technique for oxidizing graphite with K_2_Cr_2_O_7_. The authors reduced the reaction time from 5 days to 20 h, and also showed that sodium nitrate does not affect the reaction. A key feature of the GO obtained by the aforementioned method is the many times higher content of the edge oxygen-containing groups, namely carboxyls and ketones/aldehydes, with the simultaneous drastic decrease in the content of the basal plane hydroxyl and epoxy (C-O-C) groups.

Furthermore, in order to obtain GO with various oxidation degrees, electrochemical methods of oxidation of thermally-expanded graphite have been used [37,38]. Such methods are environmentally-friendly and fairly simple to implement; however, the functional composition of the oxidation products obtained in the above articles has not been studied.

Despite these results, the question of a simple, scalable synthesis method of GO with a given oxidation state (C/O ratio), a relative concentration of given oxygen-containing groups, and the absence of contaminants remains open. Furthermore, for the relationship between the parameters of synthesis and the chemistry of graphene oxide, in particular, the ratio of oxygen-containing groups on the surface and edges of the graphene layer remains unclear.

In this article, we propose a novel approach for the synthesis of GO, which allows the management of GO chemistry via the use of a combination of KMnO_4_ and K_2_Cr_2_O_7_ oxidizing agents. The novelty of the presented work is in a simple, previously-undescribed approach to the production of GO with a controlled, predetermined functional composition through the selection of the ratio of oxidants in the mixture directly in the process of GO synthesis. It should be noted that the results obtained indicate the possibility of the fine quantitative tuning of the content of basal-plane and edge-located functional groups of GO. This is an excellent opportunity for the development of the field of functionalized graphene compounds, as most of them are obtained from GO, and the possibilities of its functionalization directly depend on its initial functional composition. Using a set of spectroscopic methods, we have studied in detail the interplay between the KMnO_4_/K_2_Cr_2_O_7_ ratio in the oxidizing mixture and the composition of basal-plane and edge-located oxygen-containing groups. The structural, morphological, and optical features of the synthesized GO samples due to the changes in the chemistry of the material have also been studied via a set of microscopic methods, the laser diffraction method, and UV-Vis spectroscopy. Given all of the results, the facile method for the one-step synthesis of CMGs with the desired composition of oxygen-containing groups and optical properties is presented, opening up new possibilities for advances in the optoelectronic (e.g., organic solar cells based on rGO with covalent-bonded perovskite quantum dots) and electrochemical (batteries, li-ion accumulators, hybrid supercapacitors) applications of graphene-related materials.

## 2. Materials and Methods

### 2.1. GO Synthesis

Graphite (EG-350-80, China), H_2_SO_4_ (92%, chemical grade, Sigmatec, Moscow, Russia), HCl (37%, chemical grade, Sigmatec, Moscow, Russia), H_2_O_2_ (30%, chemical grade, Merck, Darmstadt, Germany), KMnO_4_ (chemical grade, Mosreaktiv, Moscow, Russia), and K_2_Cr_2_O_7_ (chemical grade, Mosreaktiv, Moscow, Russia) were used for the GO synthesis.

The synthesis of the GO samples was carried out according to a method based on the Hummers [32] and Rosillo-Lopez [36] methods. For the oxidation of graphite, a mixture of KMnO_4_/K_2_Cr_2_O_7_ oxidizing agents in a different ratio was used. The proportion of each oxidizing agent in the mixture was determined in accordance with the proportion of the amount required for the complete oxidation of graphite. It is known [32,36] that the complete oxidation of 1 g of graphite requires 3 g of KMnO_4_ or 7.5 g of K_2_Cr_2_O_7_. According to our synthesis method, if the KMnO_4_/K_2_Cr_2_O_7_ ratio is 20:80, then the KMnO_4_ fraction was calculated as 20 wt.% of 3 g, and the K_2_Cr_2_O_7_ fraction as 80 wt.% of 7.5 g. Thus, the complete oxidation of such a sample requires 0.6 g of KMnO_4_ and 6 g K_2_Cr_2_O_7_. A set of six samples denoted hereinafter as MC#1–MC#6 were synthesized with the KMnO_4_/K_2_Cr_2_O_7_ ratio varying from 0:100 to 100:0. Table 1 shows the composition of the samples, and the oxidant ratios used for their synthesis.

Each sample was synthesized as follows. In total, 1 g of graphite was added to a cold 80% H_2_SO_4_ and stirred on a magnetic stirrer in an ice bath (T = 4 °C). Then, the oxidizing agent or the mixture was added in small portions within 30 min. Afterwards, the reaction mixture was heated to 45 °C and stirred for two hours. After the completion of the oxidation, hydrolysis was carried out by adding distilled H_2_O to the reaction mixture, followed by adding 30% H_2_O_2_, in accordance with the Hummers method. The product was washed with 2.5% HCl with the sequential centrifugation of the sample, decantation of the solution, and dilution with a new portion of HCl until the estimated residual H_2_SO_4_ content in the sediment was less than 0.0001 wt.%. The final part of the washing was carried out with distilled H_2_O via several cycles of centrifugation using a laboratory centrifuge (SIGMA 8KS, Osterode am Harz, Germany) at 20,000× *g* acceleration, until the residual HCl content in the sample was less than 0.001 wt.%. Then, the product was passed several times through a mesh with a mesh size of 60 μm in order to remove unoxidized particles.

### 2.2. Characterization

The chemistry, morphology, and optical properties of the synthesized MC#1–MC#6 samples were analyzed by means of Fourier transformed infrared spectroscopy (FTIR), X-ray photoelectron spectroscopy (XPS), X-ray absorption spectroscopy (XAS), laser diffraction (LD), X-ray diffraction (XRD), Raman Spectroscopy, UV–Vis spectroscopy, transmission electron microscopy (TEM), atomic-force microscopy (AFM), and optical microscopy.

The Fourier transform infrared spectroscopy was carried out using an Infralum-08 IR spectrometer (InfraLUM, St. Petersburg, Russia) equipped with an attenuation of total reflectance (ATR) attachment.

The X-ray photoelectron spectra were measured on the Russian–German channel of the BESSY-II electron storage ring (Helmholtz-Zentrum Berlin, Berlin, Germany), using an ultrahigh vacuum experimental station on the beam channel [39]. The survey X-ray photoelectron spectra were measured at an excitation energy of 736 eV with a step of 0.5 eV, 200 μm slit width, and an energy pass value of 50 meV. The C 1s X-ray photoelectron spectra were measured at the excitation energy of 850 eV with the step of 0.05 eV, a 200 μm slit width, and an energy pass value of 20 meV. The as-measured C 1s spectra were further aligned with respect to the position of the reference Au 4f7/2 line (84.0 eV) and deconvoluted with the use of CasaXPS@ software (Version 2.3.16Dev52, Casa Software Ltd., Teignmouth, TQ14 8NE United Kingdom). The C 1s spectra were fitted by Shirley background and a set of one asymmetric Doniach–Sunjic function (DS) and five symmetric Gaussian-Lorentzian product functions of 70:30% ratio (GL (30)). A nonlinear least-squares routine was applied in order to achieve the best agreement between the experimental spectra and their fitting.

The XAS measurements were carried out within the range of 280–305 eV at a magic angle (48°), with a step of 0.1 eV in the total electron yield (TEY) mode realized by changing the energy of the incident photons and the simultaneous recording of the sample drain current. The obtained spectra were further subjected to the standard procedure of normalization and smoothing [40]. The samples for the FTIR, XPS, and XAS studies were prepared by the drop-casting of 25 μL of the MC#1–MC#6 aqueous suspension of 0.05 wt.% concentration onto the surface of a silicon wafer, with subsequent drying at room temperature overnight.

The size distribution of the GO platelets was estimated on the base of the LD measurements of the MC#1–MC#6 aqueous suspensions of 0.01 wt.% concentration using a Mastersizer 2000 (Malvern Panalytical, Malvern, United Kingdom) in accordance with the procedure described by Rabchinskii et al. [41].

The X-ray diffraction (XRD) analysis was carried out by applying two geometries to thoroughly investigate the structural parameters of the studied GOs. In order to acquire the detailed information on the staking order, XRD measurements with the reflection geometry were carried out using an URD-6 X-ray diffractometer (Seifert FPM GmbH, Freiberg, Germany). GO papers of ~1–5 μm of thickness were studied during these measurements. The GO papers were obtained by drying 5 mL of the corresponding GO suspensions 1.1 wt.% in concentration overnight at room temperature. At the same time, in order to study the in-plane imperfections in the layers of the studied GOs, additional XRD measurements with the transmission geometry were performed, using instrumentation for single-crystal X-ray diffraction analysis, namely a Bruker Smart Apex Duo (Bruker, Karlsruhe, Germany). For these measurements, free-standing GO films of 200–300 nm thickness with a lateral size of several millimeters were fixed on the cactus needle using nitrocellulose lacquer and placed within the aperture of the X-ray beam. The GO films were manufactured analogously to the procedure described for the FTIR, XPS, and XAS studies. The XRD patterns were acquired at different angles between the samples’ normal surface and the direction of the X-ray beam, lying within the range from 15° to 75°. Thus, the acquired sets of the 2D XRD patterns were further recalculated into 2θ data.

The Raman spectra were obtained on a Centaur U HR Raman spectrometer (Nano Scan Technologies LLC, Dolgoprudny, Russia) at an excitation length of 532 nm at several points for each sample. The samples for the Raman studies were prepared analogously to the ones for the XPS and FTIR studies.

The UV-Vis optical density spectra of the MC#1–MC#6 aqueous suspensions of 0.01 wt.% of concentration were measured using a Shimadzu-2450 spectrophotometer (Shimadzu, Kyoto, Japan) in the range of 190–800 nm, with a 1 nm step and a quartz cuvette with a 1 mm optical path.

The AFM images were taken with a Solver P47 AFM instrument (NT-MDT, Zelenograd, Russia) in the semi-contact mode at a scan rate of 0.5 Hz. Silicon TESPA-V2 probes (Bruker, Carteret, NJ, USA) with nominal resonant frequency of 300 kHz and a nominal tip radius of 10 nm were used. Samples for the AFM imaging were prepared by the drop casting of a GO water suspension with a concentration of 0.001 wt.% on a silicon substrate, and further drying at room temperature on the air. The set of three samples for each GO were studied in order to obtain statistics on the number of layers and detailed data on the morphology for the synthesized materials.

The TEM images and the corresponding selective area electron diffraction (SAED) patterns were acquired using a Jeol JEM-2100F electronic microscope (JEOL, Akishima, Tokyo, Japan), operating at accelerating voltage of 200 kV, with a point resolution of 0.19 nm. The samples for the TEM and ED studies were prepared by the deposition of MC#1–MC#6 films by dip-coating the TEM Cu grid (300 Mesh) in the corresponding aqueous suspension of 5·10^−4^ wt.% concentration.

From the moment the synthesis started and throughout it, a sample was taken every 30 min in order to study the ongoing processes using an optical microscope. The optical microscopy images were obtained by a POLAR 3 optical microscope (Micromed, Shenzhen, China). The samples for the optical microscopy were prepared by the drop casting of a 20 µL reaction mixture probe with a 20 µL of distilled H_2_O onto a glass substrate. The set of 100 images for each GO on each time point were studied in order to obtain detailed data on the synthesis process. The complete hydrolysis and washing of the samples from the components of the reaction mixture were deliberately not carried out. This made it possible to observe the processes occurring in the original layered non-exfoliated structure.

## 3. Results and Discussion

### 3.1. FTIR Analysis

**Figure 1:**
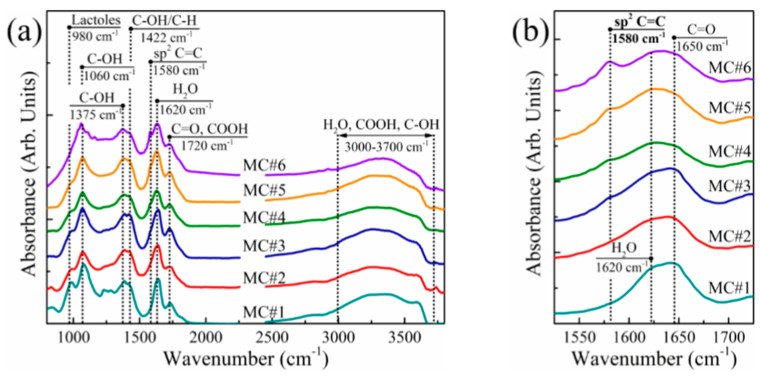
(**a**) Survey and (**b**) enlarged at 1520–1720 cm^−1^ FTIR spectra of the MC#1–MC#6 films. The spectra are vertically offset for clarity.

In order to obtain data on the chemistry of MC#1–MC#6 samples, FTIR analysis was performed. The obtained spectra (Figure 1a) were consistent with the literature data on the FTIR studies of GO [42,43,44]. At the same time, a difference in the spectra of the GO synthesized with the different KMnO_4_/K_2_Cr_2_O_7_ ratio was indicated. A distinguishable peak near *ṽ* = 1225 cm^−1^, corresponding to epoxy groups, was present only in the spectra of samples MC#1 and MC#2. Similarly, the intensity of the peaks near *ṽ* = 1060 cm^−1^ and *ṽ* = 980 cm^−1^, attributed to hydroxyls and lactols, respectively, decreases progressively from MC#1 to MC#6. This was accompanied by the reduction of the absorption band positioned at *ṽ* = 1620 cm^−1^, and is commonly attributed to the interlayer water [42]. Combined, these changes indicate a drastic decrease in the content of the basal plane oxygenic groups upon the decrease of the KMnO_4_/K_2_Cr_2_O_7_ ratio.

**Figure 2:**
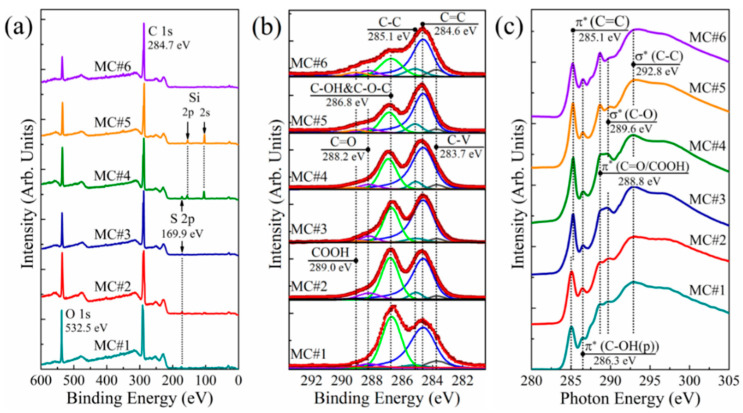
(**a**) Survey and (**b**) C 1s X-ray photoelectron spectra of the MC#1–MC#6 films; (**c**) C K-edge X-ray absorption spectra of the MC#1–MC#6 samples. The spectra are vertically offset for clarity.

On the other hand, the band at *ṽ* = 1720 cm^−1^—attributed to carboxyl groups [44]—rises, and the sharp peak at *ṽ* = 1580 cm^−1^ related to conjugated C=C bonds appears and progressively intensifies with the increase of the K_2_Cr_2_O_7_ rate in the oxidizing mixture (Figure 2b). This suggests the extension of the overall area of the unoxidized *sp2*-domains and functionalization of graphene edges with carboxyls at a higher content of K_2_Cr_2_O_7_ as an oxidizing agent.

### 3.2. XPS and XAS Analysis

The assertions made on the basis of the FTIR studies were further supported by the XPS and XAS data. Figure 2a displays the survey X-ray photoelectron spectra of the MC#1–MC#6 samples. Only the C 1s and O 1s core level signals at the binding energies of *hv* = 284.7 eV and *hv* = 532.1 eV, respectively, are observed in the acquired Survey spectra. The spectral lines at *hv* = 100.3 eV and *hv* = 150.5 eV correspond to Si 2p and Si 2s signals from the substrate, respectively. At the same time, no signals attributed to the elements of the reaction mixture, namely S 2p (*hv* = 167.1 eV), K 2p (*hv* = 293–296 eV), Cr 2p (*hv* = 575–580 eV), and Cl 2p (*hv* = 195–200 eV), can be distinguished. This points out the high purity of the synthesized GOs, with a content of impurities less than 0.1 at.%. As the O 1s core level signal is also affected by the contribution from the interlayer and adsorbed water, the detailed data on the C/O ratio as well as the composition of the oxygenic groups was obtained by the further analysis of the processed high-resolution C 1s spectra (Figure 2b).

Six peaks, corresponding to either carbon atoms in non-functionalized areas of the graphene network (peaks C-V at *hv =* 283.7 eV, C=C at *hv =* 284.6 eV, and C-C at *hv =* 285.1 eV) or carbon atoms bonded with the oxygenic groups (peaks C-OH&C-O-C at *hv =* 286.7 eV, C=O at *hv =* 288.2 eV, and COOH at *hv =* 289.0 eV) can be discerned in the C 1s spectra after deconvolution. The origin of the peak C-V is still debated, being related either to the presence of carbohydrate groups (C-H/C-H_2_) or non-terminated carbon atoms at the edges of the vacancy defects [45]. At the same time, peaks C=C and C-C are well-known components in the C 1s spectra of graphitic materials, and correspond to the sp^2^-hybridized π-bonded carbon atoms, and the carbon atoms of the distorted lattice with a single σ-bond, respectively [46,47]. The asymmetrical shape of the C=C peak is due to the screening effect of the π-conjugated system, appearing upon the photoionization of the electrons of the sp^2^-hybridized carbon atoms [48].

Three peaks—C-OH&C-O-C, C=O, and COOH—correspond to the basal-plane hydroxyls and epoxides, edge-located carbonyls, and edge-located carboxyls, respectively [49]. The presented C 1s spectra clearly demonstrate the progressive reduction of the C-OH&C-O-C peak, with the simultaneous rise of the C=O and COOH peaks upon the transition from the KMnO_4_ to K_2_Cr_2_O_7_ as a main oxidizing agent. This is accompanied by the corresponding changes in the C K-edge X-ray absorption spectra of MC#1–MC#6 samples displayed in Figure 2c. The drastic growth of the peak at *hv =* 288.8 eV corresponding to π*-resonance in the C=O and COOH groups is observed with the simultaneous diminishing of a spectral feature at *hv =* 289.6 eV, which is commonly attributed to the σ*-resonance of C-O bonds in basal-plane groups [21].

**Figure 3:**
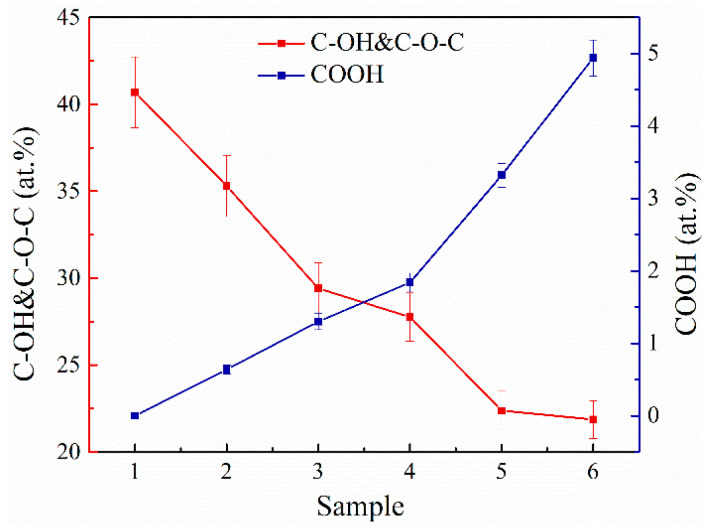
The changes in the concentration of the basal-plane (C-OH&C-O-C) and carboxyl (COOH) groups upon the change of the relative concentration of the of KMnO_4_ and K_2_Cr_2_O_7_ oxidizing agents.

For a more detailed analysis, the data of the C 1s spectra were subjected to quantitative analysis, the results of which are shown in Table 2. According to the obtained data, the amount of basal C-OH and C-O-C groups on the surface decreases almost linearly by two times, from 40.69 at.% for MC#1 to 21.87 at.% for MC#6 (Figure 3). Conversely, the number of carboxyl groups demonstrates a monotonous rise from about 0.1 at.% in MC#1 to almost 5 at.% in MC#6 with the reduction of the KMnO_4_/K_2_Cr_2_O_7_ ratio. Thus, the FTIR, XPS, and XAS data collectively point out the possibility to accurately tune the composition of the basal-plane (C-OH&C-O-C) and edge-located (C=O, COOH) oxygenic groups by the alteration of the oxidizing mixture’s composition. One of the side results of this process is the increase of the C/O ratio from 2.26 in MC#1 to the highest value of 2.92 in MC#5. A slight reduction of the C/O ratio in MC#6 and the overall still-high oxidation degree of the synthesized GOs are due to the aforementioned rise in the content of carboxyls. This oxygenic group carries two O atoms per one C atom, and thus has the strongest impact on the value of the C/O ratio compared to the moieties. Accordingly, an increase in the number of carboxyls will dominate the effect from the other oxygenic groups’ reduction in terms of the C/O ratio value, replacing its rise by diminishing. Such an effect can be seen in the case of the MC#5 and MC#6 samples: the prominent rise of the content of carboxyls from 3.32 at.% to 4.94 at.% prevails over the slight diminishing in the content of hydroxyls and epoxides from 22.38 at.% to 21.87 at.%, forcing the C/O ratio to reduce from 2.92 to 2.62 (Table 2).

### 3.3. Optical Microscopy and Laser Diffraction Analysis

**Figure 4:**
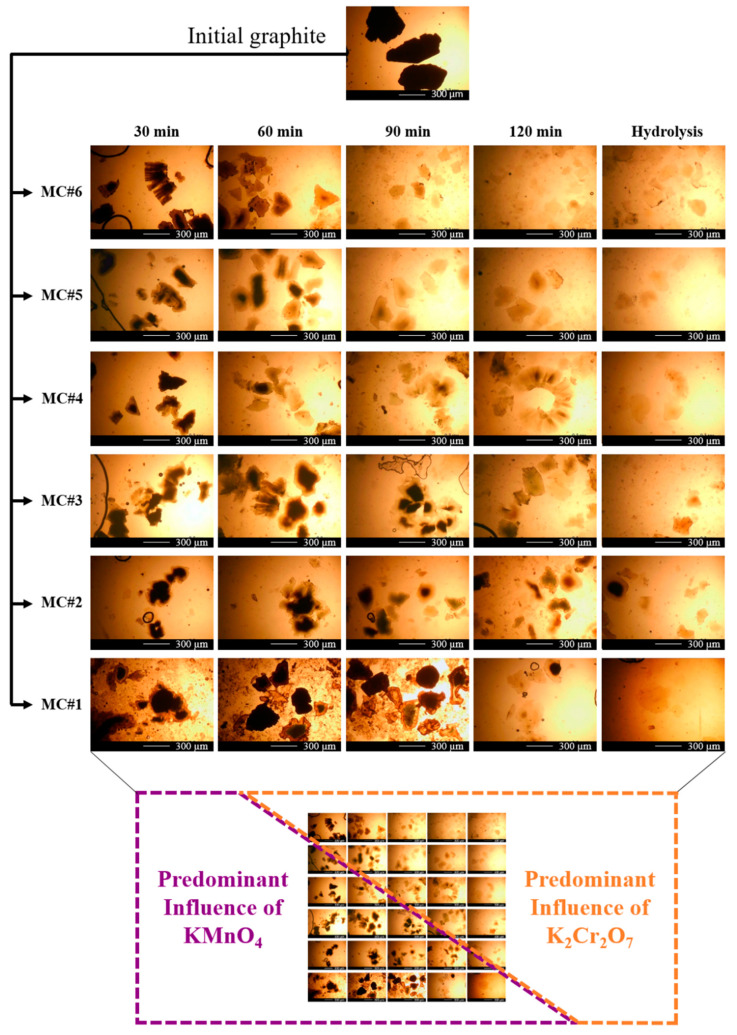
Optical images of the MC#1–MC#6 GO synthesis process every 30 min during the 2 h of the reaction time, from the initial graphite in H_2_SO_4_ to the final product before purification.

Besides the effect on the composition of oxygenic groups, the changes in the KMnO_4_/K_2_Cr_2_O_7_ ratio were also demonstrated to modify the morphology and structure of the synthesized material. Figure 4 displays the optical photos of the MC#1–MC#6 platelets at different times of oxidation and after hydrolysis. The oxidation process for sample MC#1 with pure KMnO_4_ proceeded according to the classical oxidation model proposed by Dimiev and Tour [50]. According to it, the oxidation of graphite starts from the edges, protrudes to the center, and proceeds according to the diffusion mechanism. As the particles of the used graphite are rather large (the average size is 400–500 µm), a significant amount of under-oxidized particles remain in the mixture after the end of the reaction. They are indicated by a dark core area (insufficiently oxidized graphene layers; the ‘under-oxidized’ phase) and an almost transparent shell of well-oxidized areas of graphene layers (Figure 4, MC#1, 120 min). The simultaneous presence of an under-oxidized center and well-oxidized shell results, in particular, in the break off of the GO platelets at the edges and the slight decrease of their size to 250–350 µm. This originates from the high mechanical stress arising at the core/shell interface, due to the opposition of rather densely-packed graphite sheets in the under-oxidized intercalated region, bound by van der Waals forces, and already extremely-oxidized sheets in the shell, which are inevitably repulsed by the same negative charge oxygen-containing groups on the surface of each of them.

**Figure 5:**
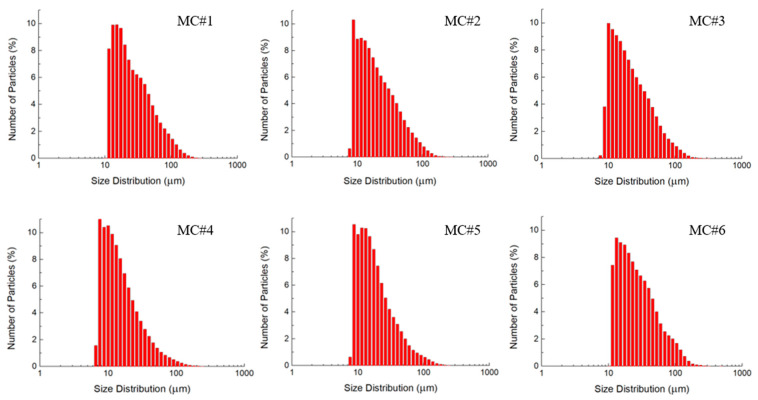
Size distribution bar charts according to the laser diffraction analysis of the dispersions of MC#1–MC#6 GO samples with equal concentration.

It should be noted that at the stage of observing the process of the reaction by means of optical microscopy, the complete hydrolysis and washing of the samples from the components of the reaction mixture were not carried out intentionally. The study of the synthesis process in dynamics by means of optical microscopy can be carried out only by observing the evolution of the layered structure, which is destroyed when the GO is removed from the acidic medium due to the dissociation of oxygen-containing groups on the surface of each layer and the Coulomb repulsion of equally-charged GO sheets. Close attention was paid to the large particles, as the differences in the ongoing processes and the type of their development are most clearly visible on them. In addition, the complete hydrolysis and washing of the samples lead to the destruction of large particles, and the average particle size significantly decreases when going from the layered to the monolayer form of GO (Figure 5).

Approximately the same situation is observed for sample MC#2, for which the fraction of K_2_Cr_2_O_7_ during oxidation is still minor compared to the fraction of KMnO_4_. In samples MC#3 and MC#4, at the initial stages, flakes with a core-shell structure were also observed. However, the shape of the particles differed significantly from the initial one. Many ragged edges of an acute-angled shape appeared, replacing rounded boundaries, and the particle size continued to decrease. In addition, a characteristic feature of these samples was the presence of a large number of expanded layered worm-shaped particles (Figure 4, MC#3, 60 min; MC#4, 120 min). The proportion of visually under-oxidized particles decreased at earlier stages (Figure 4, 120 min for MC#3 and 60–90 min for MC#4).

For the samples MC#5 and MC#6 during the first hour of the reaction, a large number of expanded layered particles were also observed. Particles with a dark center were also present in the MC#6 sample obtained by oxidation only with K_2_Cr_2_O_7_. However, a distinctive feature of these particles was the absence of a clearly defined core/shell interface. After 90 min of the process, the core practically disappeared, and the amount of visually under-oxidized particles decreased drastically. By 120 min, it was almost impossible to find particles with a pronounced dark core in samples MC#5 and MC#6, whereas the lateral size of the GO platelets decreased to 50–250 μm.

Two distinctive areas, referring to the predominant influence of KMnO_4_ and K_2_Cr_2_O_7_ on the graphite oxidation process, can be distinguished in Figure 4. They are separated by a hypothetical diagonal from the MC#5 image at 30 min to the MC#2 image at 120 min. Using such a scheme, it can be seen that the predominant effect of each of the oxidants is proportional to its fraction in the oxidizing mixture. With a decrease in the proportion of one of the components of the oxidizing mixture, its consumption occurs at shorter time intervals, and the course of the process shifts to one which is characteristic of another oxidizer, the fraction of which is higher.

The analysis of the optical images in combination with the data on the chemical analysis of the obtained samples allowed us to conclude that the mechanism of graphite oxidation using K_2_Cr_2_O_7_ differs significantly from the oxidation by KMnO_4_ [50]. The diffusion mode is not typical for the sample obtained by oxidation with K_2_Cr_2_O_7_. The dominant process is the exfoliation and refinement of graphite particles under the action of K_2_Cr_2_O_7_. Owing to these factors, the oxidation predominantly proceeds at the edges of the exfoliated graphene platelets, leading to the formation of edge-located carboxyls and carbonyls. At the same time, the formation of basal-plane hydroxyls and epoxides is suppressed, resulting in the retention of large areas of the unfunctionalized sp^2^-conjugated graphene network. However, a more detailed study of the oxidation mechanism and chemical processes occurring under the influence of K_2_Cr_2_O_7_ on graphite requires deeper research.

Figure 5 displays the LD data on the size distribution of the synthesized GOs. No significant differences in the particle size of the GO samples of different chemical composition can be observed. The size distributions for all of the samples demonstrate a single maximum in the range of 10–30 µm, with a gradual decrease in a fraction of platelets up to a size of 150 µm. However, it should be noted that particles < 1 µm are not correctly diagnosed by the LD method, which is seen by a sharp decrease in the size distribution at small values of particle sizes. This is due to the peculiarities of the mathematical processing of diffraction patterns within the framework of the model used in laser diffraction analysis of particles [43]. Small particles exhibit a less-resolved scattering pattern, with broadband maxima in the range of angles from 0 to 90°. The scattering pattern becomes a smooth curve without any detectable features in the range of lateral sizes of less than 1 μm. Consequently, it is extremely difficult to detect the presence of small particles in the diffraction patterns of polydisperse systems, even in the multimodal model. Thus, the changes in the fraction of small (100 nm–1 μm) GO platelets in MC#1–MC#6 is still under question.

### 3.4. Raman Spectra and XRD Patterns

**Figure 6:**
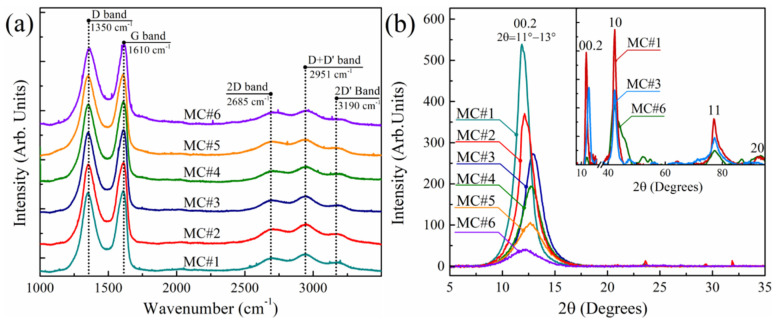
(**a**) Raman spectra of the MC#1–MC#6 samples. The spectra are vertically offset for clarity. (**b**) XRD powder patterns of the MC#1–MC#6 samples, measured within the range of 2θ from 5° to 35° using the reflection geometry. Inset: XRD patterns of the MC#1, MC#3, and MC#6 samples measured using transmission geometry. The area of 2θ = 18–35°, containing the signal from the needle used to fix the samples, is excluded. The relative intensities of the 00.2 maxima of MC#1, MC#3, and MC#6 in the XRD patterns measured using the reflection and transmission geometry are equal.

The morphological and structural features of the synthesized MC#1–MC#6 GOs were further investigated via a set of methods, including Raman spectroscopy and XRD. The Raman spectra of GOs synthesized with different KMnO_4_/K_2_Cr_2_O_7_ ratios demonstrate almost no differences (Figure 6b). Two major bands at *ṽ* = 1350 cm^−1^ and *ṽ* = 1610 cm^−1^ are presented in all of the Raman spectra, corresponding to those commonly observed in the graphene-related materials D band and G band, respectively. The former is related to lattice disorder, particularly the distortion of carbon bonds and the corrugation of the graphene net, whereas the G band refers to the in-plane stretching of the graphene lattice [51,52]. For all of the studied GOs, the I_D_/I_G_ ratio lies within the range of 0.95–0.97, with no detectable shifts in the position of the D band and G band. The position and relative intensity of the second-order bands—namely the 2D band (*ṽ* = 2685 cm^−1^), D+D’ band (*ṽ* = 2935 cm^−1^) and 2D’ band (*ṽ*= 3202 cm^−1^)—also remains unchanged. These facts suggest the absence of significant changes in the morphology of GO platelets synthesized with KMnO_4_/K_2_Cr_2_O_7_ ratio, namely the overall extent of the edges and number of wrinkles and folds [22]. However, the alterations in the nanostructure of the MC#1–MC#6 samples can also be hindered in the Raman spectra by the effect of the number of layers in the studied films, as well as the dominating contribution of the edges and oxidized areas of the GO platelets.

This is signified by a thorough examination of the structure and morphology of the MC#1–MC#6 samples via X-ray diffraction analysis. Figure 6b displays the XRD patterns of all of the GO samples. An intense and narrow maximum at *2θ* = 11° was observed in the XRD pattern of MC#1, which refers to a diffraction reflection from the (00.2) planes with a basal spacing (d_basal_) of 7.8 Å. This value is consistent with the published data for GO synthesized via the Hummers method [53], originating from the presence of basal-plane groups and coordinated interlayer water molecules. Upon the increase of the K_2_Cr_2_O_7_ ratio, the 00.2 diffraction maximum demonstrates a slight shift to higher angles, reaching 2θ = 13° for the MC#3. This value corresponds to the interlayer distance of d_basal_ = 6.8 Å. Such an evolution of the XRD pattern indicates the reduction of the interlayer spacing due to the decrease in the number of basal-plane oxygenic groups in accordance with the FTIR and XPS data. However, with the further increase of the K_2_Cr_2_O_7_ ratio, the 00.2 diffraction maximum begins to shift back to smaller angles, almost reaching the initial value of 11°. For the MC#6 sample, the 00.2 diffraction maximum is located at 2θ = 12.2°, which corresponds to d_basal_ = 7.2 Å. Besides the established absence of the restacking of the graphene layers commonly observed for the rGOs with the appearance of the typical XRD maximum at 2θ = 25–26.4° [54], the revealed inverted shift of the 00.2 maximum in the series of MC#4–MC#6 samples asserts that the boost in the number of carboxyls results in the distortion and corrugation of the graphene layers. This would explain the reverse increase of the interlayer distance, even though the number of basal-plane oxygenic moieties was reduced.

Such an assertion is supported by the analysis of the evolution of the 00.2 diffraction maximum, as well as the 2D diffraction maxima 10 and 11 signified in the XRD patterns of MC#1, MC#3, and the MC#6 samples measured by applying the transmission geometry (Figure 6b, Inset). The appearance and parameters of the asymmetrical 10 and 11 diffraction maxima in the vicinity of the hk.0 reflections of graphite (commonly denoted as hk indices) are determined by the rotational and translational disorder (‘turbostratic’ disorder) in the stacking of the hexagonal carbon layers (graphenes) [55,56]. Thus, changes in these XRD maxima allow it to signify the alterations in the morphology of the graphene layers.

In the MC#1-MC#3 samples, only proportional changes in the intensity of the 00.2, 10 and 11 diffraction maxima were observed, with no modification of their shape. Although the half-width of the 00.2 diffraction maximum remains unaltered, giving the value of an average lateral size of the coherent scattering region (CSR) for 00.2 reflections of about 6 nm, the half-width of the 11 diffraction maximum increases with the corresponding reduction of the CSR_11_ value from 9 nm to 7 nm. In turn, in the case of the MC#4-MC#6 samples, the 00.2 XRD maximum demonstrates substantial diminishing in comparison to the 10 and 11 maxima. At the same time, for these maxima, changes in the asymmetrical shape along with a significant broadening are indicated. The CSR_00.2_ and CSR_11_ values for the MC#6 samples were reduced to 2–3 nm. Such a modification of the 10 and 11 XRD maxima amid the absence of valuable changes of the lateral size of the GOs’ platelets can originate only from the appearance of nanoscale defects, such as vacancies and nanometer-sized holes.

### 3.5. Morphology: TEM Images and SAED Patterns

**Figure 7:**
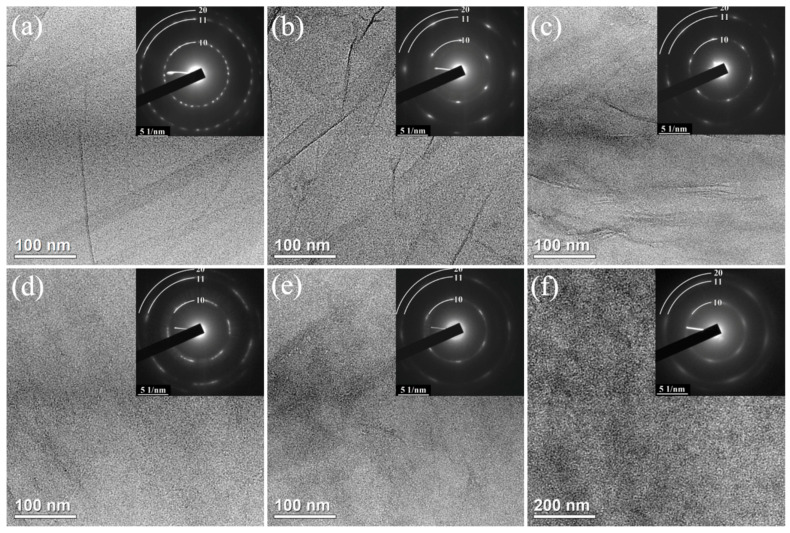
(**a**–**f**) TEM images and corresponding SAED patterns (insets) of the MC#1–MC#6 samples, respectively. The SAED patterns were acquired using a selective aperture with an 100 nm effective diameter.

The increase in the extend of structural disorder of GO sheets along the MC1–MC6 row is also evidenced by TEM studies. Figure 7 shows the representative low magnification TEM micrographs of the MC#1–MC#6 GO platelets and corresponding SAED patterns. The MC#1–MC#3 samples exhibit a continuous defect-free lamellar structure. No holes or rips are indicated in the arrays of TEM images acquired for each sample. On the other hand, the distortion of the graphene layer and progressive formation of the sub-nanometer holes can be indicated in the TEM images of MC#4–MC#6, particularly in the latter sample. Particularly, the comparison of the TEM images of the MC#1 and MC#6 samples with a higher number of areas of different contrast in the latter also hints at this suggestion.

Analogously, the ED patterns demonstrate the decrease of the structural order of the graphene sheets as a function of the KMnO_4_/K_2_Cr_2_O_7_ ratio. All of the GOs’ ED patterns are represented by one or several sets of hexagonally arranged diffraction spots. In the case of MC#1 sample, the ED pattern was acquired from the multilayer area of the sample, which is indicated by the presence of a set of hexagonal diffraction patterns rotated relative to each other. The estimated number of layers is about five or six, which coincides with the value of CSR_00.2_ calculated from the XRD patterns: six GO layers with the interlayer distance of 7.8 Å gives about 5.4 nm, comparable with the 6 nm of the calculated CSR_00.2_. For all of the other samples, the ED patterns were measured in the monolayer areas of the deposited GO layers.

Upon moving from MC#1 to MC#6, the gradual blurring of the diffraction spots was observed. In the ED pattern of MC#6, the diffraction spots almost merge into a ring-shaped diffraction pattern. Such a transformation of ED pattern is commonly attributed to rotational deformations in the structure and its nanoscale corrugation, as the imaged graphene sheets become not perfectly perpendicular to the electron beam [57]. Furthermore, diffraction spots and rings become more diffuse, and broaden. These imply the reduction of the dimensions of the defect-free crystalline regions with the reduction of the long-range order in the plane of a sheet, with the graphene layers steadily transforming to a nano-domain structure. The results of the ED analysis coincide with the conclusions made from the XRD analysis, indicating the self-consistency of such an interpretation of the changes in the morphology of GO with the increase of the K_2_Cr_2_O_7_ ratio in the oxidizing mixture.

We assert that the alterations in the morphology and nanostructure of the GO layers with the increase of the K_2_Cr_2_O_7_ ratio originates from the higher reactivity of this oxidizing agent. This results in the formation of vacancies and subnanometer holes during the oxidation, rather than the formation of basal-plane hydroxyls and epoxides observed in the case of KMnO_4_. Note that the presence of a high number or vacancies and subnanometer holes is also asserted by the growth of the relative content of C-V and C-C components in the C 1s X-ray photoelectron spectra upon the transition from MC#1 to MC#6. Apparently, the edge sites of the graphene layer at these defects are terminated by the carboxyls and carbonyls, the number of which substantially increases from MC#1 to MC#6. Owing to spatial constraints, these oxygenic groups begin to have an out-of-plane orientation [21], leading to the additional distortion and corrugation of the graphene layer. Thus, collectively, the XRD, TEM, and SAED data imply that the oxidation of GO with high K_2_Cr_2_O_7_ ratios in the reaction mixture results in the structural disorder of the graphene layers, expressed by the formation of the in-plane nanoscale defects inevitably accompanied by the out-of-plane sheet structural variations.

**Figure 8:**
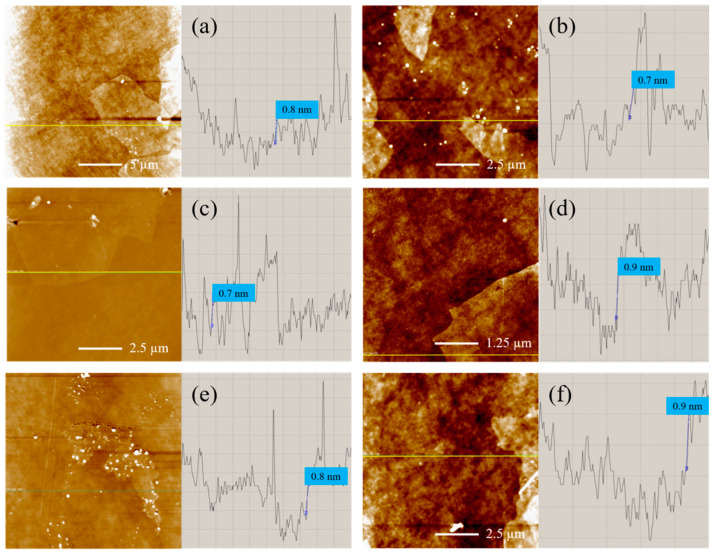
(**a**–**f**) AFM images and the corresponding height profiles of the MC#1–MC#6 GOs deposited onto the surface of silicon wafers, respectively.

Despite the discussed modifications in the nanostructure from MC#1 to MC#6, the GO platelets for all of the studied samples retain a lamellar structure. This is indicated by the acquired AFM images displayed in Figure 8. The analysis of the height profile shows that the thickness of the monolayer GO platelets—regardless the synthesis method—is 0.7–0.9 nm. The obtained values agree with the literature values [58,59,60]. Only in several samples were a few layered structures found, which was associated with the aggregation or self-assembly of two or three layers of GO during the drying process at the preparation of the samples for measurements.

### 3.6. UV-Vis Spectroscopy

**Figure 9:**
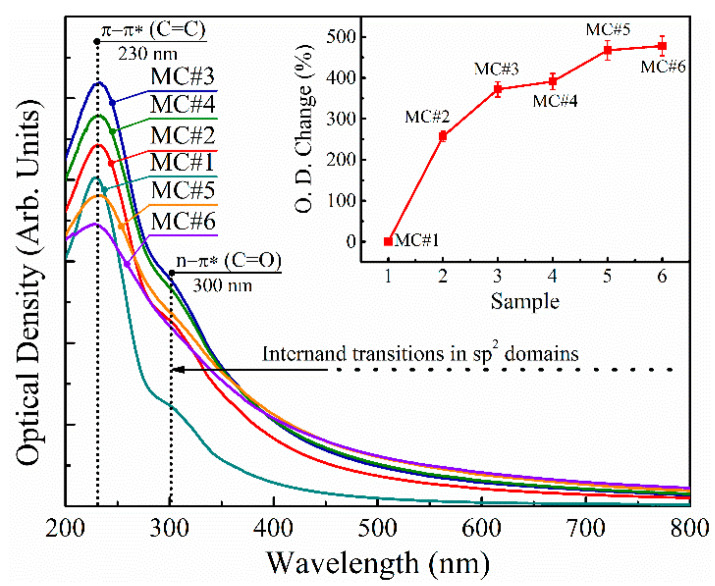
UV-Vis spectra of the MC#1–MC#6 aqueous suspensions of 0.01 wt.% concentration. Inset: the increase in the optical density in the visible range of spectra (380–750 nm) from MC#1 to MC#6.

In order to additionally investigate the effect of the oxidation with the different KMnO_4_/K_2_Cr_2_O_7_ ratios on the properties of the synthesized GOs, we performed a comparative study of the UV-Vis spectra of MC#1–MC#6 aqueous suspensions of 0.01 wt.% concentration (Figure 9). All of the presented UV-Vis spectra are governed by the main absorption peak at *λ* = 230 nm and a shoulder at *λ* = 300 nm, which is commonly attributed to the π-π* electronic transitions of C=C bonds and *n*-π* electronic transitions in chromophore C=O/COOH groups [61,62]. At the same time, a monotonously-decreasing absorption in the visible range of spectra (*λ* = 400–800 nm) is due to the π-π* interband transitions in the sp^2^-domains, the size of which regulates the bandgap [63], and thus the absorbance at a certain wavelength.

According to the measured UV-Vis spectra, MC#1 exhibits the most pronounced peak at *λ* = 230 nm, and an almost complete absence in the absorbance in the visible region. This correlates with the highest oxidation degree and content of the basal-plane oxygenic groups—hence the smallest size of the sp^2^-domains—embedded within the matrix of the functionalized graphene network. Upon the reduction of the KMnO_4_ ratio and the increase of the K_2_Cr_2_O_7_ content with the corresponding diminishing of the number of basal-plane oxygenic groups (Table 2), the intensity of the peak at *λ* = 230 nm and overall absorption in the near UV and visible regions increase. The overall absorption in the visible range continues to rise with the increase of the K_2_Cr_2_O_7_ ratio (MC#4–MC#6), resulting in an almost five-fold enhancement of the optical absorbance in MC#6 compared to MC#1 (Figure 9, inset). However, the rise of the peak at *λ* = 230 nm becomes replaced by its diminishing. Moreover, despite the more than three-fold increase in the number of the carboxyls and carbonyls from MC#1 to MC#6, the absorption band at *λ* = 300 nm—attributed to the electronic transitions in these oxygenic functionalities—also diminishes. These results make the commonly-used interpretation of the peaks at *λ* = 230 nm and *λ* = 300 nm strongly debated, in accordance with the results of Smirnov et al. [64]. We assert that both of these absorption bands have a more complex nature, not related only to optical transitions in certain chromophore groups or molecular fragments of the graphene oxide network but originating from the peculiarities of its electronic structure, particularly the electronic levels of the carbon atoms at the sp^2^–sp^3^ interfaces [65], and the formation and evolution of the localized π-plasmons and σ-excitons [66]. Hence, further studies devoted to the interplay between the chemistry and optical properties of GO and other CMGs are needed.

## 4. Conclusions

In this paper, we demonstrated for the first time an approach for the management of GO chemistry via the use of KMnO_4_/K_2_Cr_2_O_7_ oxidizing agents at different ratios. The almost linear dependence of the content of the basal-plane hydroxyls/epoxides and the edge-located carbonyls/carboxyls on the KMnO_4_ to K_2_Cr_2_O_7_ ratio was indicated. Combined with almost two-fold change in the number of hydroxyls/epoxides and the three-fold change in the number of carbonyls/carboxyls upon the transition from the KMnO_4_ to K_2_Cr_2_O_7_ as a predominant oxidizing agent, the proposed approach is asserted to allow the synthesis of GOs with the desired composition of oxygenic groups. The modification of the GO chemistry is suggested to arise from the difference in the mechanisms of the oxidation of graphite with K_2_Cr_2_O_7_ and KMnO_4_ indicated by the studies by Optical microscopy during the oxidation process. In comparison to the well-known diffusion-based mechanism of graphite oxidation by KMnO_4_, the reactions lying behind the graphite treatment with K_2_Cr_2_O_7_ require further studies.

Beside the alteration of the chemistry, no significant changes in the lateral size, morphology, or defectiveness of the synthesized GOs were indicated. Only the nanoscale corrugation of the GO layers upon their oxidation predominantly by K_2_Cr_2_O_7_ was demonstrated by means of TEM and ED analysis. This effect is likely to arise due to the presence of vacancy defects and the low number of basal-plane groups, which at a large content compensate the out-of-plane distortions of the graphene lattice induced by each other. The studies of the UV-Vis spectra of the synthesized GOs, having a different content of chromophore carbonyl/carboxyl groups and π-conjugation rate, also revealed several contradictions with the current model of the optical absorbance in GO. These results point out the predominant role of the GO electronic structure with the influence of excitonic and plasmonic states on the optical absorption of GO, rather than the effect of optical transitions in the chromophore groups.

Given these results, a facile and easily-scalable method for the low-cost bulk-quantity production of GOs with the desired chemistry and optical properties is proposed, opening up new horizons for the application of graphene-based materials in a wide field of applications.

## Figures and Tables

**Table 1:** Composition of oxidizing agents used in the synthesis of the GO samples.

№	K_2_Cr_2_O_7_, g	KMnO_4_, g	K_2_Cr_2_O_7_:KMnO_4_
MC#1	0	3	0:100
MC#2	1.5	2.4	20:80
MC#3	3	1.8	40:60
MC#4	4.5	1.2	60:40
MC#5	6	0.6	80:20
MC#6	7.5	0	100:0

**Table 2:** C/O ratio, and the composition of the functional groups and carbon atoms in the graphene network (in at.%) in the MC#1–MC#6 films derived from the analysis of the deconvoluted C 1s X-ray photoelectron spectra.

Component	C-V	C=C	C-C	C-OH & C-O-C	>C=O	COOH	C/O Ratio
**Binding Energy (eV)**	283.7	284.6	285.1	286.8	288.2	289.0	
MC#1	5.83	47.9	2.11	40.69	3.47	<0.10	2.26
MC#2	2.55	49.94	4.59	35.30	6.98	0.64	2.29
MC#3	2.58	56.26	4.73	29.41	5.18	1.84	2.61
MC#4	3.02	57.84	4.93	27.77	4.74	1.70	2.81
MC#5	2.83	58.09	8.09	22.38	5.29	3.32	2.92
MC#6	5.99	52.44	8.34	21.87	6.42	4.94	2.62

## Data Availability

The data presented in this study are available on request from the first author.
